# Using photovoice to generate solutions to improve food security among families living in remote Aboriginal and/or Torres Strait Islander communities in Australia

**DOI:** 10.1186/s12889-024-18200-x

**Published:** 2024-03-13

**Authors:** Emma Chappell, Ellie Chan, Caroline Deen, Julie Brimblecombe, Yvonne Cadet-James, Marita Hefler, Emma Stubbs, Megan Ferguson

**Affiliations:** 1 School of Public Health, The University of Queensland, Herston, QLD 4006 Australia; 2 Menzies School of Health Research, Charles Darwin University, Casuarina, NT 0811 Australia; 3 Central Australian Aboriginal Congress, Alice Springs, NT 0871 Australia; 4 Apunipima Cape York Health Council, Bungalow, QLD 4870 Australia; 5 Department of Nutrition, Dietetics and Food , Monash University, Level 1, 264 Ferntree Gully Road, Notting Hill, VIC 3168 Australia; 6 Indigenous Education and Research Centre, James Cook University, Bungalow, QLD 4870 Australia

**Keywords:** Food security, Photovoice, Aboriginal, Indigenous, Remote health

## Abstract

The right to food security has been recognised internationally, and nationally in Australia by Aboriginal Community Controlled Health Organisations. This study aims to explore food (in)security and solutions for improvement of food security in remote Aboriginal and/or Torres Strait Islander communities in Australia, from the perspective of caregivers of children within the context of the family using photovoice. Participants took part in workshops discussing participant photographs of food (in)security, including solutions. Themes and sub-themes with associated solutions included traditional food use, sharing as a part of culture, the cost of healthy food, energy and transport, and housing and income. Community leaders used these data in setting priorities for advocacy to improve food security in their communities.

## Background

Prior to colonisation, Aboriginal and Torres Strait Islander peoples consumed a nutrient-dense diet.[1] Since then a history of dispossession, genocide, and discriminatory food policy has led to disrupted food security.[2] A definition of food security for remote communities of Australia has been developed by members of Aboriginal communities through the Good Food Systems: Good Food For All project:

‘Food security for us is when the food of our ancestors is protected and always there for us and our children. It is also when we can easily access and afford the right non-traditional food for a collective healthy and active life. When we are food secure we can provide, share and fulfil our responsibilities, we can choose good food knowing how to make choices and how to prepare and use it.’[3].

A commonly used international definition by the Food and Agriculture Organization (FAO) is that food security is when “all people, at all times, have physical, social, and economic access to sufficient, safe and nutritious food that meet their dietary needs and food preferences for an active and healthy life”.[4]The FAO estimates 29.6% of people worldwide experience moderate or severe food insecurity, with the highest prevalence (60.9%) experienced by people in Africa.[5] In high-income countries such as Canada and the United States, Indigenous peoples experience a high burden of food insecurity, at 25–69% [6–9] compared with non-Indigenous households at 9% (Canada) [10] and 10.5% (US).[11] Young children and their caregivers are at particular risk of physical and mental health impacts of food insecurity.[12–14]

Today Aboriginal and Torres Strait Islander peoples demonstrate resilience to food insecurity through, for example, sharing relationships [15,16] and use of traditional foods.[15–17] However, food insecurity is reported to be higher in remote Aboriginal and Torres Strait Islander populations (31%) compared with the rest of Australia (4%),[18] and research shows the prevalence may be underreported, with remote food insecurity noted to be as high as 76%.[19] In Australia, remoteness is classified according to basic access to services into five categories: major cities, inner regional, outer regional, remote and very remote.[20] Here we refer to communities defined as either ‘very remote’ or ‘remote’, as remote. There are 1187 ‘discrete Aboriginal communities’ in Australia, defined as having at least a 50% Aboriginal and/or Torres Strait Islander population, usually having community-managed housing and/or infrastructure, and may be serviced by a local school, health clinic, council office and shop/s.[21] 75% of these communities have populations of less than 50 people; 17% have a population of over 1000.[22]

Income is lower and unemployment higher for Aboriginal and Torres Strait Islander peoples in remote communities than anywhere else in Australia.[23,24] There are 200 stores serving the 1187 remote communities, and these are commonly community-owned and governed.[25] Household food cost is consistently reported to be higher in remote communities than non-remote areas.[26–30] Food cost remains higher overall in remote communities for reasons including the high cost of freight, and the limited buying power of remote retailers compared with urban supermarket chains.[25,26] Housing issues also contribute to food insecurity. Overcrowded housing accounts for over 50% of housing in remote areas,[31] while 36% of dwellings have major structural problems and 24% lack basic facilities for preparing food, washing clothes, bedding or people, or sewerage facilities.[32]

Food insecurity in remote Australia has been well described, including studies in remote Northern Territory and South Australia that describe food (in)security cycles coinciding with fortnightly pays where management strategies include relying on long-life staples (e.g., flour and sugar) in the ‘off-pay week’ as well as cultural practices such as use of traditional foods and a sharing economy.[15–17,33,34] Other management strategies include ways to minimise the need to share and consuming takeaway foods,[15,34] selling re-packaged products and carefully planning household food purchases.[16] There has been little work that has primarily focused on the perspectives of caregivers of children in describing the experience of food insecurity and solutions to address this. Whilst the Good Food Systems: Good Food For All study explored solutions to improve food security from a community perspective,[35] solutions have not been explored at a household or individual level in remote communities.

The right to food is a human right,[36] codified in international law.[37,38] Specific rights relating to Indigenous Peoples’ cultural rights and traditional food collection activities are included in the United Nations Declaration on the Rights of Indigenous Peoples (UNDRIP) to which Australia is a signatory, and which inform the Voluntary Guidelines to Support the Progressive Realization of the Right to Adequate Food in the Context of National Food Security.[39,40] The UNDRIP also outlines the rights to self-determination, to participate in decision making, and to develop priorities, strategies and programs.[41] This demonstrates the need for Indigenous peoples to be active partners and decision makers, including in research involving them.

**Fig. 1:**
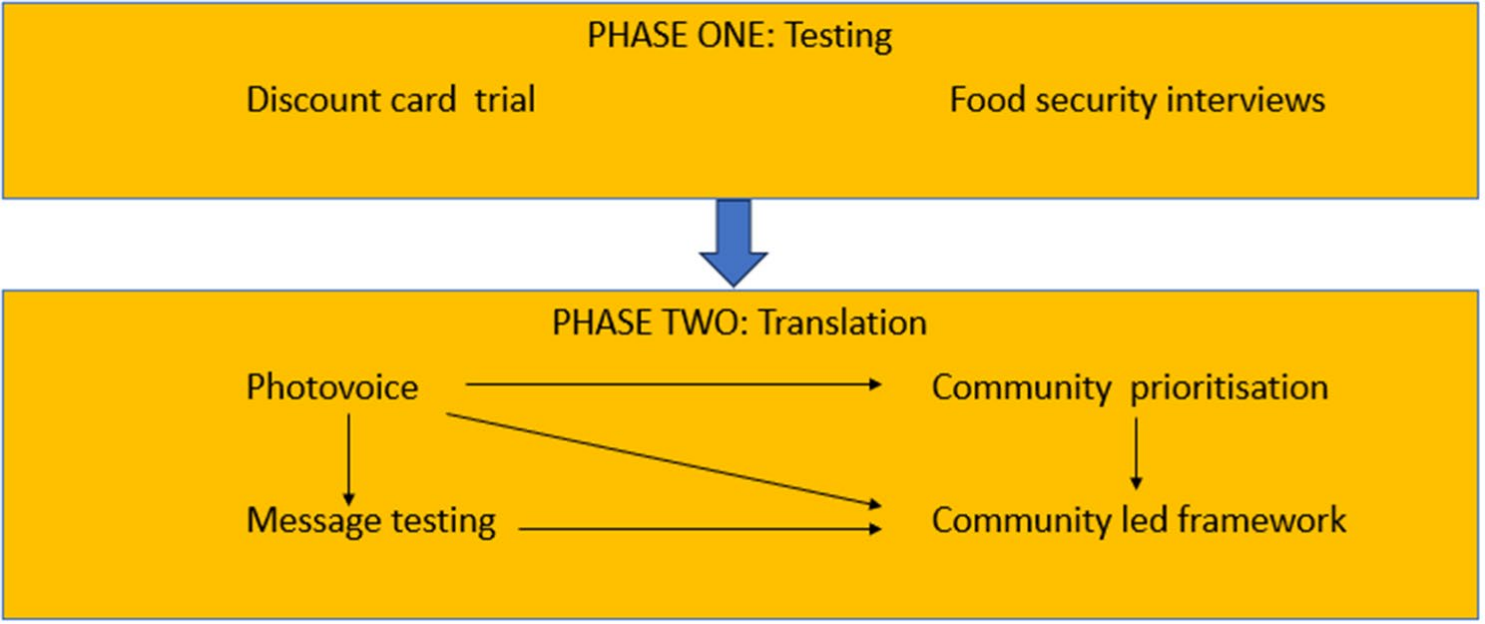
Remote food security project phases

Apunipima Cape York Health Council (Apunipima) and Central Australian Aboriginal Congress (Congress) are Aboriginal Community Controlled Health Organisations (ACCHO) governed by elected, mostly Aboriginal and/or Torres Strait Islander boards of directors. Both have identified food security as a priority for action, including for research, and have released position statements related to this.[42,43] The study reported here is nested in the Remote Food Security Project (Fig. 1), which was co-designed by Apunipima and Congress.[44] The project includes Aboriginal and Torres Strait Islander peoples throughout the governance structure: Chief Investigators, working groups, implementation team and Community Advisory Groups.[44] The implementation team included ACCHO-employed project managers (one Aboriginal, one non-Indigenous) and Research Assistants (both Aboriginal). Local Community Researchers were employed in each community. In phase one of the larger project, the ACCHO invited ten Cape York and Central Australian communities to be part of the project. Four communities were purposively selected to receive the strategy of a discount card that reduced the cost of healthy foods. Eligible participants were pregnant women and parents and carers of Aboriginal and/or Torres Strait Islander children under five. The discount card was delivered in a three-to-six month trial whereby eligible participants could receive 30% discount on healthy food purchases to a cap of AUD$80–120 depending on number of eligible participants per household, by swiping a discount card at the local community store.[44] The larger project had established relationships in these communities, including with Community Advisory Groups. The implementation team had relationships with these groups and with clinics, child and family centres and councils. In three of the four communities, English is spoken as a second or subsequent language to traditional languages (with English the primary language in the fourth).

Photovoice is a participatory tool that engages participants to take photographs of their communities’ strengths and concerns, participate in critical dialogue, and disseminate the results for policy action.[45] Apunipima identified photovoice as appropriate to explore solutions to improve food security, based on using photovoice for health promotion in Cape York, which found the method increased community participation and understanding of health issues. Photovoice has been used on various topics with Aboriginal and Torres Strait Islander populations within Australia,[46–48] including in an urban setting to address food security.[49] Internationally, a United States study engaged with parents of young children around food security, to lead to advocacy for policy action at the national level.[50] As far as the authors are aware, photovoice has not been used to explore food (in)security in a remote context in Australia with the target population.

Building on seminal work to describe food (in)security and solutions,[15,16,34,35] the objective of this paper is to explore food (in)security and solutions to improve food security in remote Aboriginal and/or Torres Strait Islander communities from the perspective of caregivers of children within the context of the family using photovoice. Our goal was to explore solutions rather than just describing an issue. To explore the issue prior to focusing on solutions our method prompted both issues and solutions. Thus, the objective of the paper reflects an exploration of both food security and food insecurity, referred to as food (in)security.

## Methods

### Theoretical approach

The Remote Food Security Project broadly and this photovoice study undertake a co-design approach to research, with reflection on best-practice guidelines for research with Aboriginal and Torres Strait Islander communities and working within research values and priorities set by ACCHO.[44] Photovoice draws on principles of participatory action research (PAR), particularly through engaging participants in data analysis and decision making and dissemination strategies. [51–56] PAR comes from a place of critical inquiry; which aims to address power differentials and create social change, and encompasses different paradigms including social justice, equity and feminist lenses.[57]

### Study planning

Photovoice, part of phase two of the larger Remote Food Security Project,[44] was facilitated in the four strategy communities. Phase two focused on translation of findings into community-identified solutions to improve food security. Community Advisory Groups and Community Researchers were employed to help with recruitment and implementation planning.

### Recruitment

Purposive recruitment for photovoice, led by project managers, occurred in the strategy communities, targeting participants, or those eligible to be, of phase one of the overall project. Recruitment, which took place over two to three weeks, aimed to achieve maximum variation for employment status, carer type (e.g., parent, grandparent) and gender.

### Workshop protocol

Three workshops were conducted over a four-week period in each community. The protocol was developed by the first author with project managers (E.Chan, CD) and a working group of experts on food security, public health, and policy, including Aboriginal and Torres Strait Islander members. The first author, and in one community, the senior author, facilitated the workshops with the project managers. Participants were reimbursed with a AUD$20 electricity credit voucher for each workshop, decided by Community Advisory Groups to be an appropriate method and value.

The first workshop included sharing findings from phase one with participants. Participants were provided with digital cameras and given the photography prompt ‘What makes it easy or hard to get food for your family?’ Participants spent approximately two weeks taking photographs, with the implementation team assisting where required.

At the second workshop, participants selected photographs for discussion. Participants, individually or in pairs, were asked about their photographs, using questions adapted from the SHOWeD tool (Table 1),[45] which is commonly used in photovoice projects and includes prompts on solution development.

**Table 1:** Questions used in photovoice workshop 2

SHOWeD question [45]	Adapted question used in workshop 2	Additional prompts/examples
What do you See here? What is really Happening here?	What story do you think this photo is telling?	
How does this relate to Our lives?	How does this [this situation/story about…] affect your family or your community?	
	What are your memories about this?	e.g., what are your memories of collecting traditional foods with your family?
Why does this problem or strength exist?	Why does this situation, concern, or strength exist?	Can be reworded e.g., why does this happen, why do you think this happens e.g., why do you think people run out of food? If relevant could also ask ‘why does this happen for some people but not for other people?’
What can we Do about this?	What can we do about it? How should this problem be solved?	How could this problem be solved by community, by us, by government?
		Further probing questions may be used to get more detail about any answer
Questions asked in workshop 3		
• Is there anything missing from this story? (ask this after each category) • Are any of YOUR photos under the wrong heading? • Is there anything we’ve misunderstood? • If any one or two of these could get more attention from the others, which would it be, which is the most important for your community? • Ask for permission to use photographs publicly, if appropriate– refer to consent forms.		

For the third workshop, photographs were placed on a felt mat, organised by researchers into draft themes. Researchers provided summaries of each theme, based on data from the second workshops, and participants, individually or in pairs, were asked to verify the placement of or move their photographs, verify or modify the summaries, add to or modify the themes (Table 1), prioritise themes, and were asked if they would like to consent to share their photographs publicly.

### Data collection and analysis

Photographs were selected by participants, guided by the photography prompt. Workshops two and three were voice recorded and scribed on butcher’s paper. The research team reviewed the scribed and audio data along with the photographs to develop draft themes for presentation at workshop three. The photograph checking process (Table 1) formed the start of the first step of Braun and Clarke’s six step data analysis process (familiarisation, initial code generation, searching for themes, reviewing themes, defining themes and writing up).[58]

The research team initially became familiar with the data through being present at, listening to, engaging with and scribing workshop data. Then, professionally transcribed photovoice workshop data from workshops two and three was analysed using NVivo software (Release 1.7, QSR International). The first author led the data analysis, reading all transcripts while listening to the recordings, making corrections and listing potential codes. A codebook was developed using the participant-checked themes as parent codes, with the summaries modified by participants contributing to additional codes. The codebook was checked by three more researchers including two Aboriginal researchers on the implementation team. All transcripts were re-read and coded, with additional codes added to the codebook. Codes were sorted into potential themes. Candidate themes with their data were reviewed and a thematic ‘map’ was generated. Summaries for each theme were developed, including data and quotes. Themes were given names and were presented in order of community strengths, then community priorities as voted by photovoice participants. Two versions of a thematic map, and all the summaries with data and quotes were shared with the research team, who provided feedback.

### Researcher positionality

Three authors are Aboriginal (YCJ, CD, ES) and five including the lead researcher are non-Indigenous. We acknowledge the history of non-Indigenous research doing research ‘on’ and ‘for’ Aboriginal and Torres Strait Islander peoples; this study focused on working *with* ACCHO in a codesign process where these organisations have set the priorities for research and selected photovoice as a method. The study operates through two-way learning throughout each stage of the process, aiming to prioritise Aboriginal and Torres Strait Islander voices. This has been continued throughout the research by, for example, prioritising participant needs and preferences by passing the workshop facilitator role from the first author to project managers (CD, E.Chan) who had established rapport with participants through working with them for over a year in the earlier phase of the larger project. This continued in data analysis by including Aboriginal and/or Torres Strait Islander peoples throughout the analysis process, from participants checking initial photovoice themes, to Aboriginal members of the implementation team playing key roles in the steps of thematic analysis.

## Results

Twenty five participants (24 female and one male), 13 from Cape York and 12 from Central Australia, were recruited and participated in the first workshops (Table 2). Of these, 19 participated in the second workshops and 15 in the third workshops. Each workshop in each community had one to two participants (we originally planned for one or more workshops of up to 10 participants per community but on direction of participants it was necessary to run workshops for smaller groups of pairs or for individuals). Most participants were employed (*n* = 14). Twenty-two were parents and three were grandparents of children under five.

**Table 2:** Characteristics of photovoice participants at workshops

		Community			
	All communities	A	B	C	D
Workshop 1					
Total participants per community, n	25	7	6	5	7
Females, n (%)	24 (96)	7 (100)	5 (83)	5 (100)	7 (100)
Employed, n (%)	14 [56]	2 [26]	3 [50]	3 [60]	6 (86)
Parents*, n (%)	22 (88)	7 (100)	5 (83)	4 (80)	6 (86)
**Workshop 2**					
Total participants per community, n	19	4	5	5	5
Females, n (%)	19 (100)	4 (100)	5 (100)	5 (100)	5 (100)
Employed, n (%)	11 [58]	1 [25]	2 [40]	3 [60]	5 (100)
Parents*, n (%)	16 (84)	4 (100)	4 (80)	4 (80)	4 (80)
**Workshop 3**					
Total participants per community, n	17	4	4	5	4
Females, n (%)	17 (100)	4 (100)	4 (100)	5 (100)	4 (100)
Employed, n (%)	10 [59]	1 [25]	2 [50]	3 [60]	4 (100)
Parents*, n (%)	14 [48]	4 (100)	3 (75)	4 (80)	3 (75)

*remainder were Grandparents

There were four overarching themes identified, including sub-themes, which were priorities for improving food security: (1) cultural practices that support food security, including traditional food use and sharing as a part of culture, (2) the cost of living, including the cost (and availability) of food and other essential items, cost and availability of energy (gas or electricity), and cost of transport, (3) income and employment, and (4) housing and infrastructure (Table 3). Each theme or its subthemes had associated photographs contributed by participants. Management strategies and solutions were identified for all themes, except for the ‘cultural practices support food security’ theme, which only had solutions in the ‘traditional foods’ subtheme where participants described ways this theme was further strengthened. There was considerable overlap between themes and subthemes (Table 3).

**Table 3:** Themes, subthemes, descriptions and links

Theme/subtheme	Description	Links with
**Cultural practices support food security**	Comprises ‘Traditional foods’ and ‘Sharing as a part of culture’	
Traditional foods	Traditional foods were accessed seasonally and prepared using traditional methods. Types of traditional foods included native lizards, turkey, kangaroo, emu, pig, geese eggs, prawns, crab, mud shells, long shell, turtles, stingray, fish, dugong, and native fruits. Traditional foods were often served with foods prepared from store foods such as damper (prepared from flour).	• Sharing as a part of culture • All cost of living sub-themes
Sharing as a part of culture	Participants talked about contributing to communal resources, sharing or borrowing resources (e.g., food, electricity credit, cars, fuel, money), sharing responsibility for shopping, cooking (including traditional food preparation), or childcare, or sharing knowledge.	• Traditional foods • Income and employment • Housing and infrastructure • All cost of living subthemes
**Cost of living**	Comprises ‘The cost (and availability) of healthy food and essential items’, **‘**The cost and availability of energy’ and **‘**The cost of transport’	• All themes and subthemes
The cost (and availability) of healthy food and essential items	The cost (and availability) of, e.g., fruits and vegetables, meat, milk, eggs, bread, heaters, baby nappies (diapers), and baby formula	• All other cost of living subthemes • Traditional foods
The cost and availability of energy	The expense (and availability) of mostly pre-paid electricity, but occasionally gas.	• All other cost of living subthemes • Traditional foods • Income and employment • Housing and infrastructure
The cost of transport	The cost of transport (personal and community) within and outside communities.	• All other cost of living subthemes • Traditional foods
**Income and employment**	Social security payments, budgeting money, benefits of employment and things you need for employment.	• Sharing as a part of culture • Housing and infrastructure • The cost and availability of energy
**Housing and infrastructure**	Overcrowded housing, house maintenance.	• Income and employment • Sharing as a part of culture • The cost and availability of energy

Some results related to being parents and carers or more often generally as part of a family unit. All themes relate to this in some way, for example collecting traditional foods with family members, sharing food and other resources with family members, and ensuring there is enough food and electricity for the family. The desire to live in housing that was not overcrowded related to a participant wanting the best life for their children. Management strategies for dealing with cost-of-living pressures included prioritising feeding children before feeding adults.

### Cultural practices support food security

#### Traditional foods

**Fig. 2:**
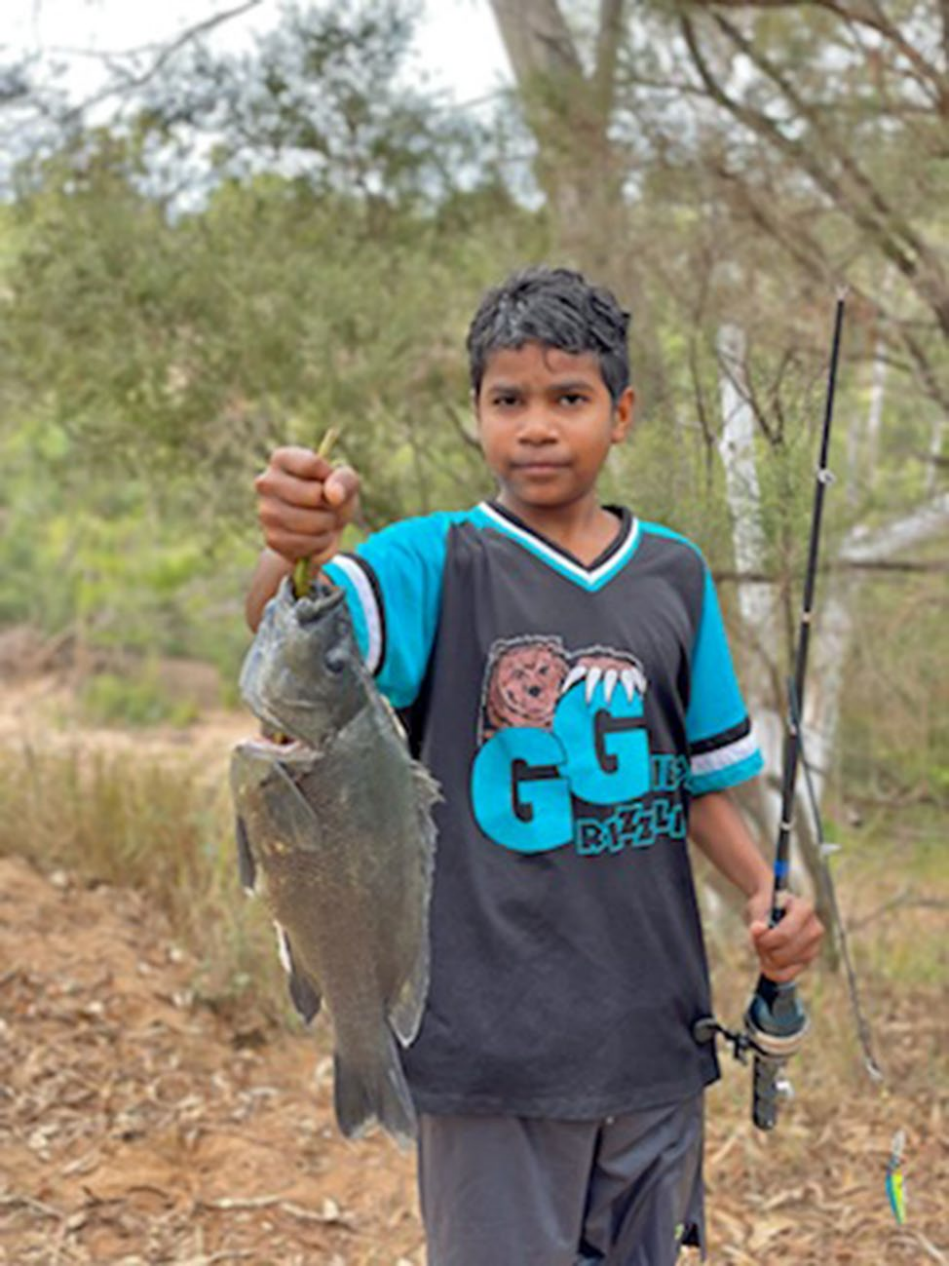
Traditional foods. © Denise Yam, Kowanyama. Used with permission

Participants described traditional foods (Fig. 2) as being free and acting as a safeguard against food insecurity though resources for accessing traditional foods were often needed, for example, cars. Benefits for food security included having money left over to spend on other resources like electricity. In addition to being protective against food insecurity, traditional food use had other benefits including being satisfying, ‘healing’(B4) and good for health, providing a chance to connect with family and a spiritual connection. Traditional food was collected ‘even when we got food in the fridge.’ (D6).

*Solutions.* Existing solutions in some communities that participants felt supported traditional food procurement included fish-washing facilities, community feasts hosted by the rangers (managers of local bushland and/or waterways) and a community bus to take people out on Country.^1^

#### Sharing as a part of culture

**Fig. 3:**
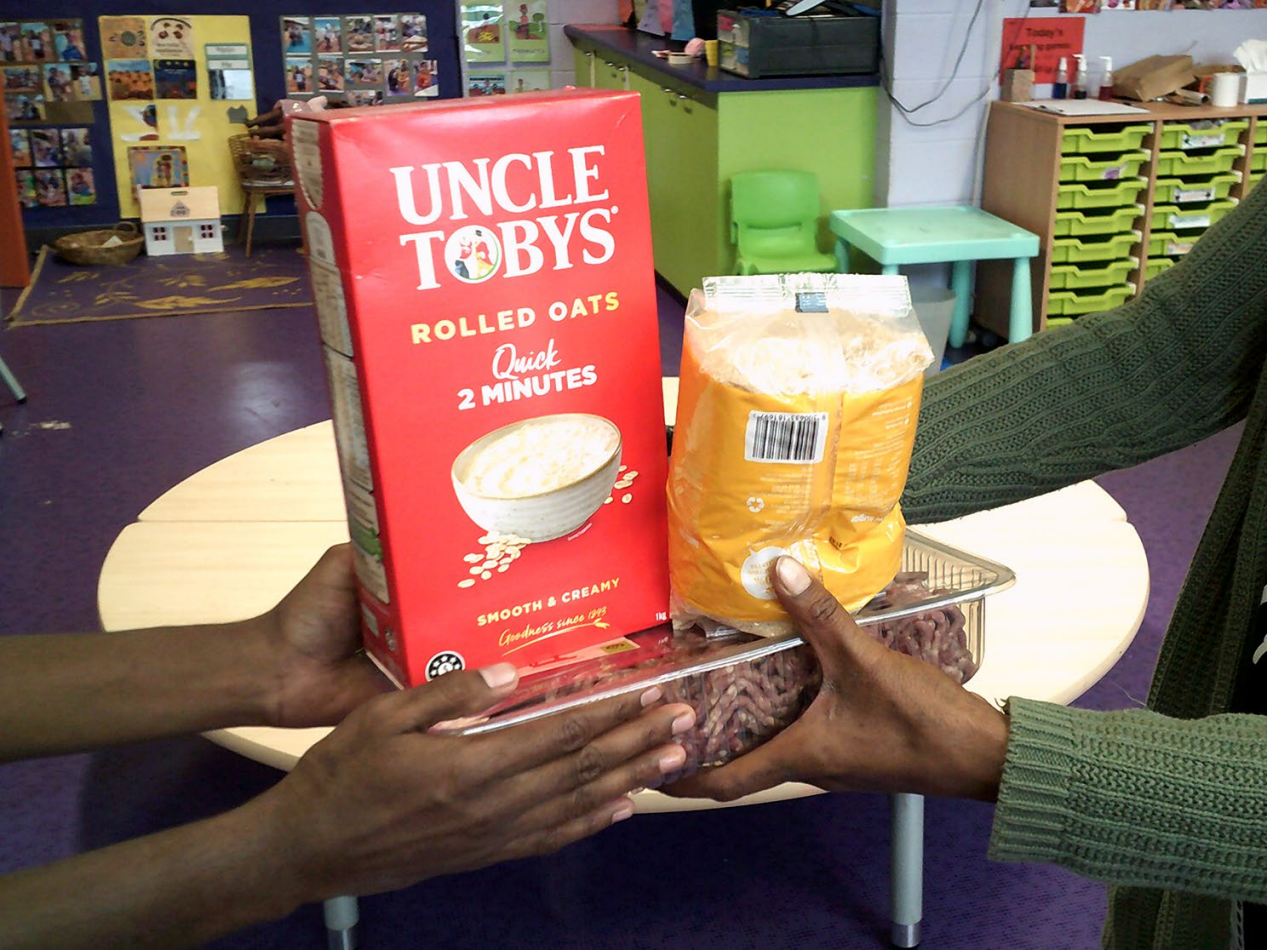
Sharing as a part of culture. © Nikkita Sampson, Yuendumu. Used with permission.

Sharing food and other resources (Fig. 3) was often described as ‘easy’, as a part of life. Participants described the intergenerational sharing of knowledge, from knowledge of traditional foods to how to navigate social security. Grandparents were described as being supported and providing support, helping raise children and themselves being cared for.

Many participants expressed a sense of pride in being ‘kindhearted’ (C4), able to help family members. Declining or being unable to share had emotional impacts for participants. Participants described how expectation of sharing can also make things hard when there are limited resources due to cost-of-living pressures. Resources are often stretched for people ‘who are not working or don’t get enough money’ (D7), when extended family come to stay, and when housing is overcrowded. Some participants spoke about needing to limit sharing to keep enough for their children.

### Cost of living

Participants spoke about the cost of living in remote locations as well as competing priorities for money including food and essential items, energy (gas or pre-paid electricity), transport and phone credit. ‘I don’t know if it’s because we’re remote or if it’s… yeah, maybe it’s because we’re remote. But it’s just, it’s yeah, really expensive living here.’ (A6).

#### The cost (and availability) of healthy food and essential items

**Fig. 4:**
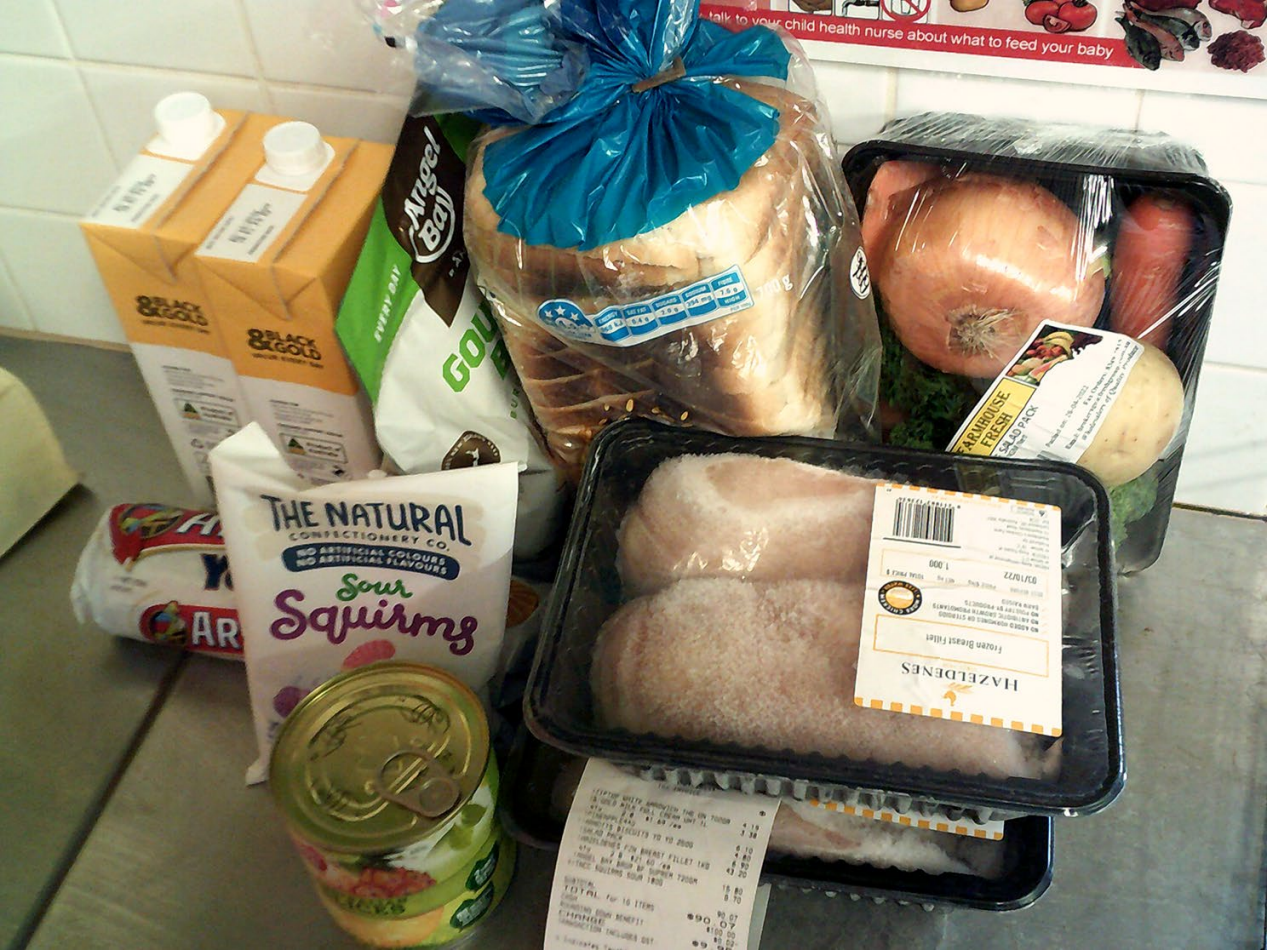
‘9 items for $90’– The cost of food. © Nikkita Sampson, Yuendumu. Used with permission.

Participants described that healthy food and other essential items in communities were expensive (Fig. 4). Participants in one community described food being more expensive in the wet season. Participants frequently compared the cost of purchases from the community store, and how long the shopping lasts, with shopping from major supermarkets in the closest towns (often many hundreds of kilometres away). One participant noted:

‘When we got no car to go into town, we go and do our shopping [in this community], and how much money we spend here, and it must be just a couple of day’s shopping, and we run out… See, when we go into town, we do more shopping. That shopping lasts us ‘til next payday.’ (C2).

Some participants noted times where the community store would run out of fresh foods, and that the quality of fresh foods was lower in the community stores, acknowledging impacts of weather and distance.

*Management strategies.* Participants said shopping at the major supermarket in the nearby town meant they did not need to choose the cheapest option and would have money left over for fuel and electricity, and that they could ‘get healthier foods’ (A6) and a better variety of food and other essential items. Some noted significant expense associated with fuel or freight when shopping this way.

Participants noted four other ways to manage the cost of healthy food and essential items. They described buying or cooking in bulk, buying and cooking cheap foods (e.g., frozen vegetables, cheap meat cuts, tinned foods, noodles or flour for damper), adults prioritising feeding children, and accessing food from services in the community such as schools and youth services.

*Solutions.* Many participants spoke of the need for decreasing healthy food and essential item prices in communities; ‘If they lower the prices down here at the shop… That’s when we’ll have more food.’ (C2) Participants spoke about lower prices in the community meaning people could stay within their community to buy healthy food and essential items.

Many participants referenced the discount card that was tested in phase one of the study (5–9 months previously). It was helpful because it ‘made the price go down a bit’ (D1) and helped save money for other cost-of-living expenses. Participants appreciated that the discount was on healthy food only, meaning they could ‘spend it in good ways. Not junk food, just get healthy food.’ (D6), and some cited using the card frequently.

Other solutions mentioned to improve the cost of food and essential items included online shopping, introducing a major supermarket to the community, education on ‘how to buy the right type of food,’ (B4) enterprises such as community gardens, butchers or abattoirs, and expansion of an existing community meals program.

#### The cost and availability of energy

**Fig. 5:**
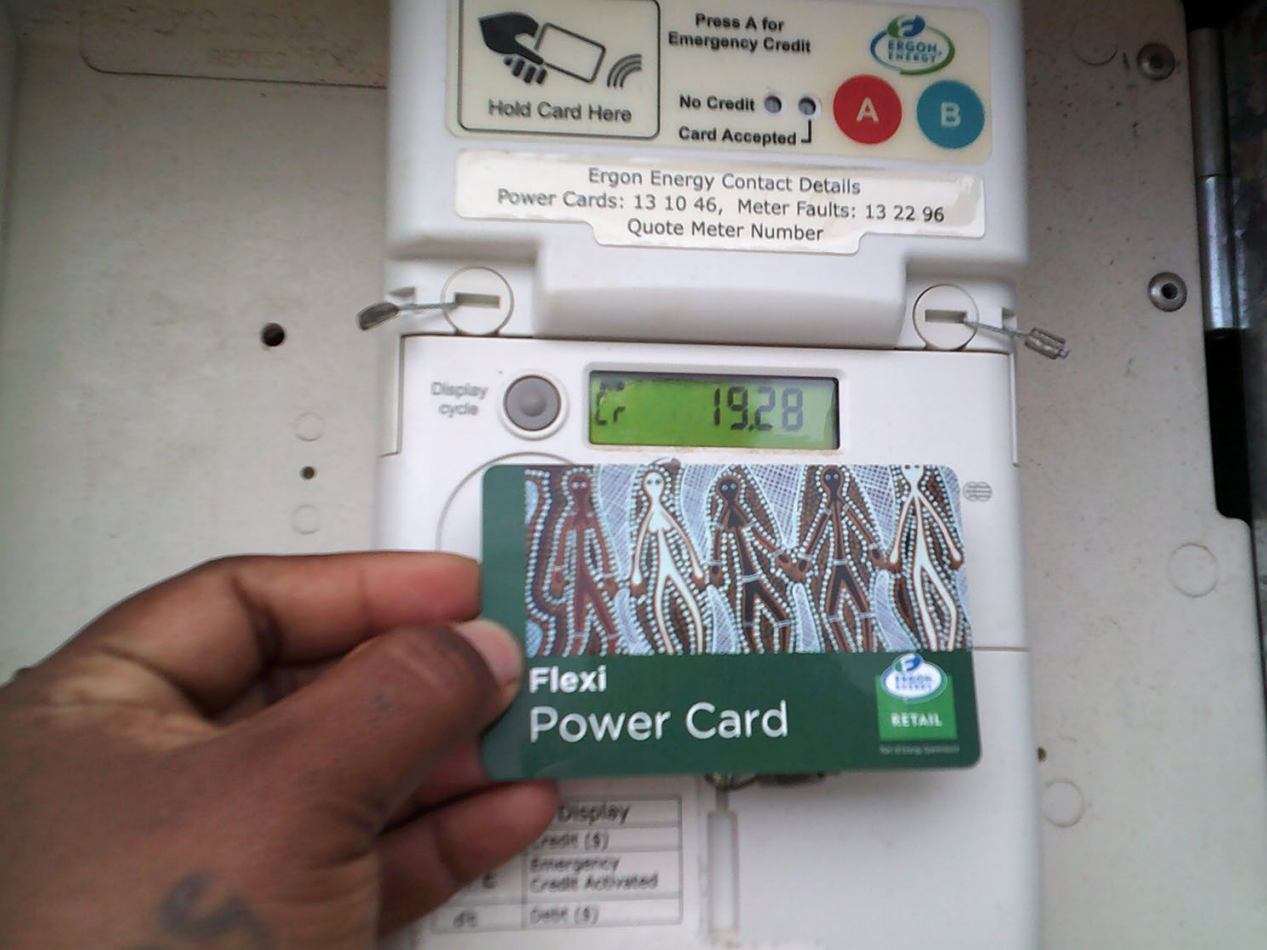
The cost and availability of energy. © Dawn Grainer, Kowanyama. Used with permission.

Participants said energy (mostly pre-paid electricity, but occasionally gas) was expensive and ran out quickly (Fig. 5). Participants described the cost of energy as competing with the cost of food, and that energy was needed for food preparation.

Participants described losing access to electricity due to running out of household credit or due to occasional community electricity outages typically associated with storms, which also impacted EFTPOS operation at the store or store closures, reducing access to food. Participants talked about factors that meant they ran out of household electricity faster, particularly use of heating or cooling:

‘Like when I used to put [AUD$] hundred in, that’ll take me two weeks. Now that it’s winter and we’re using heater, it takes like a couple of days for the power [to run out].’ (C5).

Energy was also linked with transport, with lack of transport making it harder to get to the shop to add electricity credit; with employment, with not working making it harder to afford energy; and with overcrowding, with one participant noting electricity credit was easier to manage when living in a home with just their immediate family.

*Management strategies.* Participants managed the high cost of electricity and gas in various ways, including sharing the expense between household members and energy-saving strategies. Many participants spoke about using the fire for cooking or warmth, as both a way to save energy and as a strategy when electricity or gas ran out, for example when the store is closed: ‘That’s a bit hard. Sometimes we make fires, sleep outside. Just wait for the next day.’ (D1) Other management strategies included asking family for help adding electricity credit including at times, asking family in nearby towns to add credit for them when the store in the community was closed, making meals from damper or tinned foods, and one participant described extending an electricity cord between two houses.

*Solutions.* The most common, existing ‘solution’ to improve energy security was talked about in the Cape York communities (but not in Central Australia, where this existing solution did not exist) whereby regular rebates were received for electricity credit^2^, though it was noted not all access this. All agreed it should remain to maximise energy security. Participants suggested managing electricity was easier when it was previously automatically deducted from social security payments, and as a result, never running out of electricity credit. One participant mentioned solar power as a long-term solution voiced, but not yet actioned, by their local Aboriginal Corporation.

#### The cost of transport

**Fig. 6:**
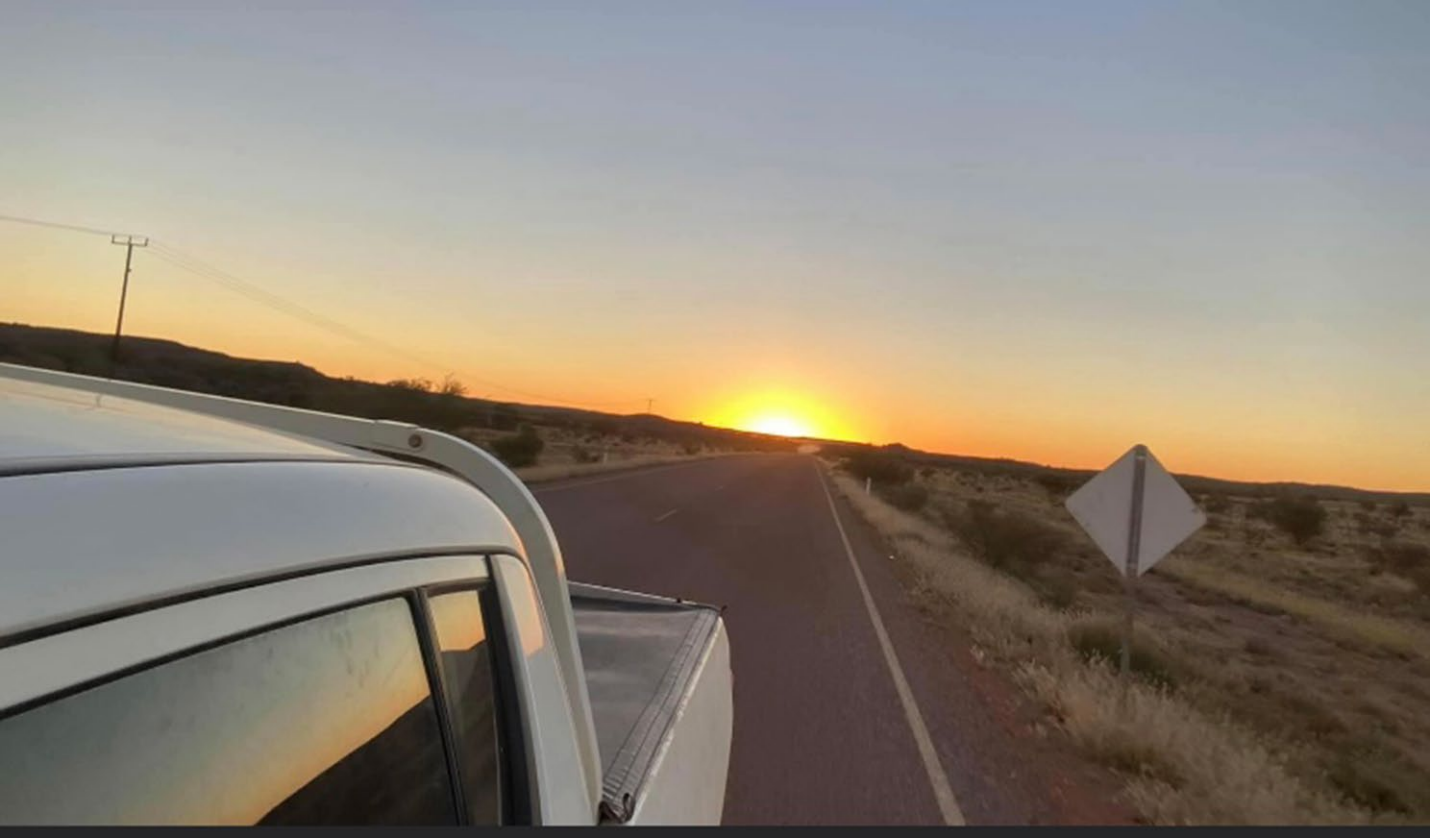
The cost of transport. © Photovoice participant. Used with permission

Participants spoke about how having access to a car contributed to food security, including increased access to traditional foods, and stores inside and outside of the community (Fig. 6). Participants spoke about food and transport as competing costs, particularly in one community where there was poor road condition and increased wear and tear on the car: ‘If I buy tyres, well, me and my family will stay hungry.’ (C2) Participants in one community talked about the expense of a bus that transported people into town. Participants talked about fuel being expensive, and that fuel was cheaper in towns.

*Management strategies.* Management strategies for transport included borrowing cars from family members, and closely monitoring fuel cost and volume compared with distance driven, as this participant describes when talking about where to procure traditional foods: ‘So you look at the fuel, you look at the fuel price, like the fuel, how much fuel you have in your car, and then you look at the distance where to go.’ (D3).

*Solutions.* Participants talked about having community or store-owned buses to transport people to the store. Participants also mentioned a bus (or a less expensive bus fare) to take people into town, and to access traditional foods.

For one community where participants talked a lot about poor road condition, the obvious solution was upgrading the road but they also felt that this had been lobbied for with no result.

### Income and employment

**Fig. 7:**
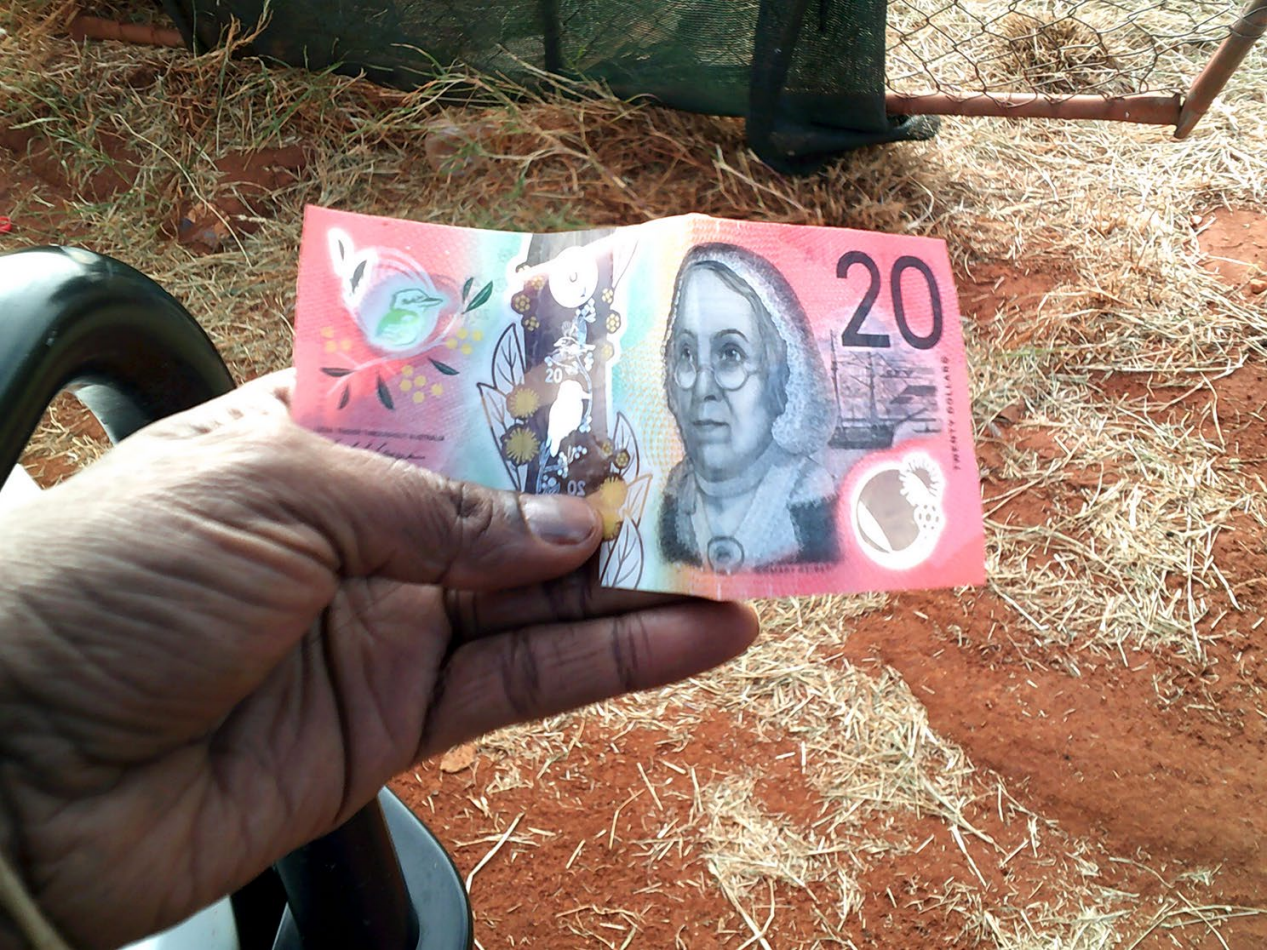
Income. © Andrea Tasman, Yuendumu. Used with permission

Participants often spoke about social security payments being inadequate, or not frequent enough (Fig. 7). One participant talked about the off-pay week as ‘drag week.’ (A6) Participants spoke about different social security payment types providing more income than others, with some payments better supporting food security.

**Fig. 8:**
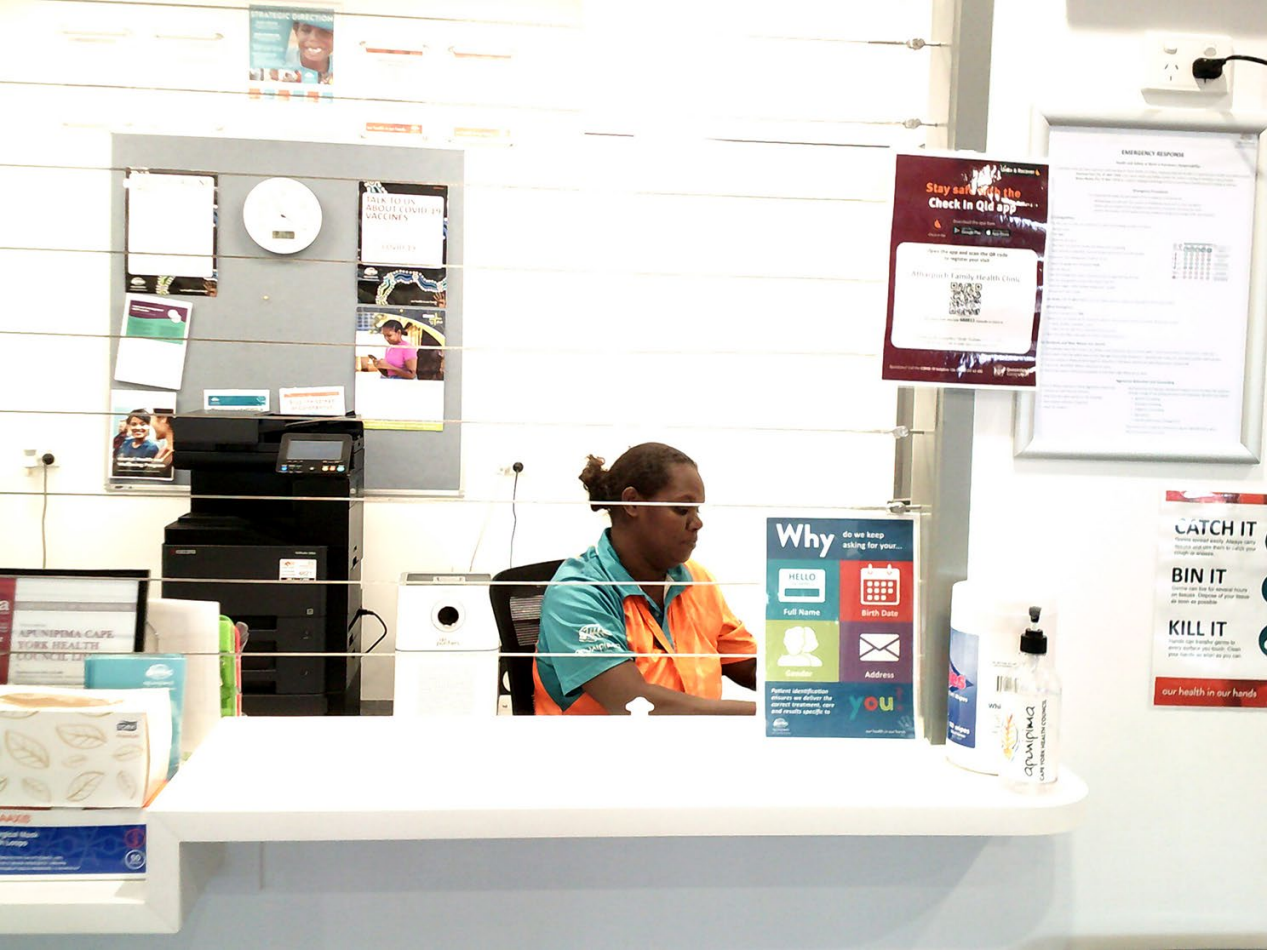
Employment. © Celestine Daniel. Used with permission

When asked about running out of money before the next pay, one participant said ‘Only the people who work find it easy.’(D7) Participants spoke about jobs as making it easier to save money for expenses, to buy food, and share food with others, and having less need to ask others for help (Fig. 8).

‘Although some families don’t eat three meals a day because they can’t afford it. If they’re not, you know, if not working full-time, how can you, yeah.’ (C3).

One employed participant described how food security looked for them: ‘Well we just do shopping every fortnight, and I buy food that can last until next pay’ (C5), and participants spoke positively about being able to share freely.

Participants noted that to get a job, having an education helped, and you needed to pass a criminal history check, and have a working with children’s card, and a form of identification (which all incur costs and are not straightforward to obtain in remote communities).

One participant talked about how some people ‘don’t budget money’ (C2). Some participants talked about ‘people spend[ing] their money wrong way too for alcohol and the bad things, you know’ (B4), including alcohol, gambling and cigarettes.

*Management strategies.* Many participants talked about budgeting or saving money as a way they manage, or intend to manage, their income, which enabled participants to buy food throughout the pay cycle, and purchase fuel for trips for traditional foods. Saving money was made easier for one participant by being able to shop in the less expensive nearby town and living in a house with just her immediate family.

*Solutions.* One participant talked about increasing income as a solution, relating this to the short-term supplement that existed as a Covid-19 response:

‘Maybe put more money in the Basic[s] card stuff. Like what they did, they put [AUD]$250 in the Basic[s] card for everyone. Maybe they should do that more [often].’ 3 (D6)

Participants in one community talked about social security payment regularity as an (existing) solution. Participants had identified that opting to split their parenting payment from their main social security payment could mean being paid twice, rather than once, in a fortnight. One participant who wasn’t aware of this said it ‘would have made life so much easier’ (A6) when they received this payment previously. One participant suggested the Basics Card was ‘really helpful’ (C2) for budgeting, another suggested some people would prefer to have cash available.

Participants suggested creating more employment and training opportunities, though one participant noted ‘we’ve been saying this for a long time.’ (B1) Participants mentioned an existing, voluntary store-based income management scheme and proposed budgeting classes as a solution.

### Housing and infrastructure

Participants related overcrowded housing with food insecurity. One participant spoke about how living with family and having ‘a lot of people in the house coming and going’ (A6) made it hard to keep leftover food for their baby. This participant described the benefits of now living in her own home with just her partner and child, including food lasting longer, and being able to save money. Participants in one community said not having a fridge was common in their community, and this made it harder to store food.

**Fig. 9:**
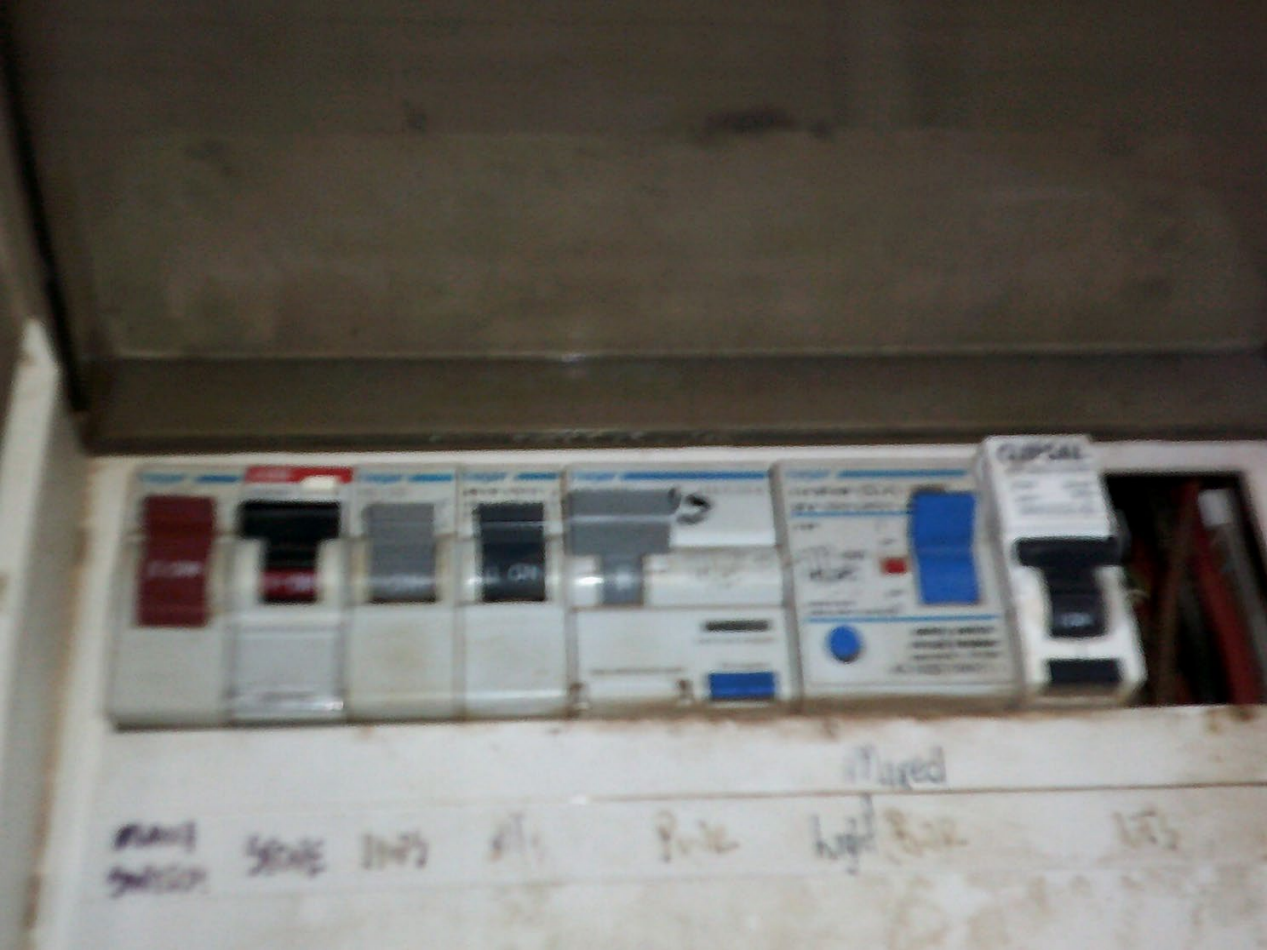
Housing infrastructure. © Photovoice participant, Ltyentye Apurta. Used with permission

Participants also talked about house maintenance as being important for food security (Fig. 9). One participant said ‘you’ve got to wait months or weeks for [the electrician] to come out, even for emergency thing.’ (C5).

*Management strategies.* Participants spoke about managing in overcrowded housing by setting limits on sharing. A participant without a fridge noted using a freezer to store frozen vegetables and using harder (dry store) vegetables like pumpkin and potato. The management strategy for a house maintenance issue where the gas stove was not available included using electric appliances.

*Solutions.* Participants suggested solutions to housing could include advocating to government to build more houses ‘because maybe there’s a lot of people waiting for a house.’ (A4) One participant cited a family member being 99th on the waitlist, and another noted her family members waiting nearly ten years for a house.

## Discussion

This study aimed to explore food (in)security and solutions to improve food security in remote Aboriginal and/or Torres Strait Islander communities from the perspective of caregivers of children within the context of the family using photovoice. Whilst some results related specifically to being caregivers of children, more often results related to the broader context of the family structure, but always it was from the *perspective* of caregivers of children. The study described priorities for food security including cultural practices supporting food security (traditional food use, sharing as a part of culture), cost of living (including cost of food, energy, and transport), income and employment and housing and infrastructure. These are consistent with previous findings in remote Aboriginal and Torres Strait Islander communities[15,16,34,63] and urban communities [64] with energy security observations included in the more recent of these.[16] Remote community residents access electricity through pre-paid credit which impacts them when electricity runs out (and the limited emergency credit available at that point is used) and the shop is closed. This impacts food security through access to cooking and storage equipment, some of which participants described managing with (e.g., through cooking outside on the fire rather than inside on the stove) but some of which cannot be managed such as the operation of fridges and freezers.

### Cultural practices support food security

The strength of Aboriginal and Torres Strait Islander culture in supporting food security has been demonstrated previously.[15,16] In the remote Northern Territory of Australia, it has been shown that 89% of households accessed traditional foods at least fortnightly, with 40% of food insecure households accessing traditional foods.[17] The sharing of food or resources among family members has been well documented in remote Australia.[15,17,34] In the study reported herein participants describe the emotional impacts of sharing or not sharing, which reflects a complex system where people are socially bound to one another, through commitments, obligations and a reciprocity that is sometimes delayed. This system has been described for example in Warlpiri culture (in Central Australia),[65] and in two diverse communities in Central Australia and Far North Queensland [66] and is explained by the people of the Anangu Pitjantjatjara Yankunytjatjara lands (also in Central Australia) as a concept based on reciprocity and kindness, ‘ngapari ngapartji’.[67] The food security definition,[3] and the human rights frameworks and instruments[36–40] mentioned earlier point both to the right of Indigenous peoples to access traditional foods, and also the right to food security generally. This study has demonstrated that whilst food insecurity forces people into situations that draw on traditional foods and cultural sharing practices, it also puts pressure on those practices. The rights relating to cultural practices can only be properly upheld when food security is achieved. Sharing relationships are easier when there is enough to go around; traditional food use is easier when there are adequate resources: while cultural practices support food security, they are also supported by food security.

### Management strategies

This study showed the diverse ways Aboriginal and Torres Strait Islander peoples manage with food insecurity, through relying on cultural practices and also through resourceful practices not previously documented such as extending electricity cords between houses to provide electricity when credit runs out. The study described management strategies previously captured, such as buying and cooking cheap foods like noodles and damper [15,34] and also noted the limitations of strategies such as procuring foods from supermarkets in nearby towns [16,34] as coming with expenses associated with fuel, freight and/or ‘wear and tear’ on the car. Management strategies in this study are consistent with both remote and urban studies in Aboriginal and/or Torres Strait Islander populations that also described filling up on cheaper, carbohydrate based foods and relying on family when resources were stretched.[15,16,34,64]

### Solutions

This study focussed on community-driven solutions (noting Booth et al. also briefly explored *suggestions* for improving food security in remote communities).[16] The final SHOWeD question asked ‘what can we do’ with prompts on what the government, research partners or community could do. A range of solutions were provided by participants in relation to cost of food, energy, and transport, income and employment and housing and infrastructure. These included a broad focus on ‘decreasing food cost’ as a priority and identifying existing (or previously existing) solutions such as electricity rebates in Cape York, and social security payment amount or regularity. Increased income, based on participant experience of the social security supplement^ii^ was echoed by other Australian residents, that the supplement was useful for accessing basic needs and making social security ‘a liveable amount.’[68] Some solutions around food cost– online shopping and increased transport into town - were at odds with participants also articulating their preference for shopping in their own community. New solutions that were specific to participants’ communities included community-run enterprises like butchers or abattoirs to decrease food cost, different methods to pay for electricity, solar power, more employment and training opportunities, and budgeting classes.

Some of the solutions identified by participants were reflected in the 2019 parliamentary committee pricing inquiry into remote stores and the Australian Government response to this.[69] The Australian Government response was not supportive of subsidies as a means to decrease food costs.[25] Internationally, there have been evaluations of subsidising food cost for food insecure Indigenous communities through freight or retail subsidies.[70,71] Evidence from these studies suggest a system of accountability and food price monitoring is needed to ensure consumers receive such subsidies.[70,71] The government response to the inquiry mentions a competitive grants scheme for feasibility studies for microgrids (solar power) for remote communities and suggests solutions to improve the reliability of energy. The government responded to the recommendation for support for local food production as a means to increase food security, by noting existing government grant schemes. Significant concerns have been raised however in relation to limited implementation of the recommendations and lack of action to address systemic failure, infrastructure, capacity building and community control; [72] something the current remote food security policy process must address.[73]

### Solution development through next steps

A strength of this study was in describing priorities for food security using first-hand participant experiences and having accompanying photographs describing solutions from the perspective of caregivers of young children. This study contributes to the ‘collective voice’ of the broader Remote Food Security Project (Fig. 1). First-hand lived experiences, photographs and solution suggestions from parents and carers of children under five were key to informing community-designed solutions to improve food security in this population.

In a community prioritisation meeting following the photovoice study in each community, community leaders reviewed photovoice data, with other findings from phase one of the project, and developed priorities and solutions. Photovoice participants were invited to participate in this workshop, and three participated. Following this, a knowledge exchange with representatives from ten study communities reviewed these results, and developed a community-led framework and an advocacy plan to improve remote food security. Community consultation work is ongoing with these communities, with photovoice results (photographs, summaries and key quotes) returned to Community Advisory Groups to be used in further advocacy work.

### Limitations

A limitation of this study is that the workshop facilitators were not speakers of Aboriginal languages. In an earlier phase of the study, where interviews on food security were conducted, Community Researchers from each community assisted with explaining in language where needed. However, participants declined to have the Community Researchers present at their food security interviews, some informally citing privacy concerns. As photovoice participants were a similar cohort, and also due to the limited availability of the Community Researchers, the photovoice workshops did not have another Aboriginal language speaker present. This may have been limiting, in that participants may not have been speaking in their first language, so some nuance and meaning may have been lost. It did however remove the barrier of sharing sensitive personal stories with someone who was known to them and their family.

Another limitation of this study was the low representation of male caregivers. The one male participant who was recruited did not go on to participate in workshops two and three. The recruitment strategy was to invite phase one participants, who were also almost all female, and then expand to other eligible participants who did not participate in phase one. In both phase one and photovoice, the research team was all-female which may have contributed to lower male participation.

## Conclusion

The strength and resilience of Aboriginal and Torres Strait Islander peoples in managing food insecurity has been identified through cultural strengths and management strategies. Participant-identified priorities (cost of living, housing and income) and solutions related to these have potential to contribute to advocacy through the next steps of the project. Solutions included broadly ‘reducing food prices’, as well as more specific novel solutions like community-based enterprises and building on existing solutions such as the energy rebate available in Cape York. Photographs and stories from parents and carers of children under five have given a unique ‘lived experience’ perspective to the next steps of this project towards advocacy to improve food security.

## Data Availability

The datasets generated and/or analysed during the current study are not publicly available due to the ethics approval granted but are available from the corresponding author on reasonable request and with ethics approval.

## Abbreviations

ACCHO: Aboriginal Community Controlled Health Organisation
Apunipima: Apunipima Cape York Health Council
Congress: Central Australian Aboriginal Congress
FAO: Food and Agriculture Organization
UNDRIP: United Nations Declaration on the Rights of Indigenous Peoples

## Footnotes

1 ‘Country is the term often used by Aboriginal peoples to describe the lands, waterways and seas to which they are connected. The term contains complex ideas about law, place, custom, language, spiritual belief, cultural practice, material sustenance, family and identity.’ [59].

2 Rebates on electricity of AUD$372.20 per year (GST inclusive) are available to customers of certain payments from Services Australia, including holders of low income cards, and can be accessed through electricity credit cards for members of remote communities that use these.[60]

3 BasicsCard quarantines a portion of social security recipients’ incomes to be spent on a card that can be used at selected outlets.[61] In 2020 the Australian Government introduced a short-term Covid supplement (initially AUD$550 per fortnight, then reduced to AUD$250) to be paid to recipients of select social security payments.[62]
